# Effects of fermented unconventional protein feed on pig production in China

**DOI:** 10.3389/fvets.2024.1446233

**Published:** 2024-07-31

**Authors:** Haoxuan Sun, Zipeng Jiang, Zhimin Chen, Guohua Liu, Zexue Liu

**Affiliations:** ^1^Cofco Joycome (Jilin) Co., Ltd., Songyuan, China; ^2^Guangdong VTR Bio-Tech Co., Ltd., Zhuhai, China; ^3^School of Biology and Biological Engineering, South China University of Technology, Guangzhou, China; ^4^Key Laboratory for Feed Biotechnology of the Ministry of Agriculture and Rural Affairs, Institute of Feed Research, Chinese Academy of Agriculture Sciences, Beijing, China; ^5^COFCO Wuhan Meat Product Co., Ltd., Wuhan, China

**Keywords:** antinutritional factor, animal nutrition, animal health, gut health, feed

## Abstract

Unconventional protein feeds, characterized by low nutritional value, high variability, and poor palatability, have limited their application in swine production. Fermentation technology holds the key to addressing these shortcomings. Given the ban on antibiotics in China, the inferior quality of imported pig breeds, and long-term dependence on imported soybean, the prospects for fermented unconventional protein feeds are promising. This paper delves into the common types of fermented unconventional protein feeds, factors influencing the fermentation process, the mechanisms by which they enhance swine health, and the challenges and prospects of fermented feeds, offering theoretical insights for the future development of the feed industry.

## Introduction

China is the largest pork producer globally and pigs hold a significant position in the national economy (1). An ancient Chinese saying, “Pigs and grain ensure the world’s peace,” underscores the importance of swine in China. However, the swine industry in China is currently facing several challenges, including the reliance on 90% imported foreign breeds, long-term dependency on imported soybean, and a government policy banning antibiotics. Most of the pigs raised in China are foreign breeds like “Duroc,” “Yorkshire,” and “Landrace,” which, despite their high reproductive rates and economic benefits, have inferior meat quality compared to indigenous Chinese breeds. The shortage of protein resources and the long-term reliance on imported soybean have limited the development of the livestock and aquaculture industries. In response, the Ministry of Agriculture and Rural Affairs of China formulated the “Plan for Reducing and Replacing Corn and Soybean Meal in Feed” in 2021, proposing comprehensive measures to explore new protein feed resources such as single-cell protein (2) and insect protein (3), thereby reducing dependence on imported soybeans. Intensive livestock production in China provides a high yield of pork to meet the food demands of the population, but it heavily relies on the extensive use of antibiotics. The use of antibiotics leads to the spread of antibiotic-resistant genes in the pig gut microbiota, which can be transmitted to the environment and humans, causing antibiotic resistance in both animals and humans (4). In 2019, the Ministry of Agriculture and Rural Affairs issued a notice to completely ban the use of antibiotics. In the face of the antibiotic ban, the Chinese feed industry needs excellent alternatives, such as antimicrobial peptides (5, 6), essential oils, and other natural bioactive compounds (7), as well as prebiotics (8).

**Figure 1:**
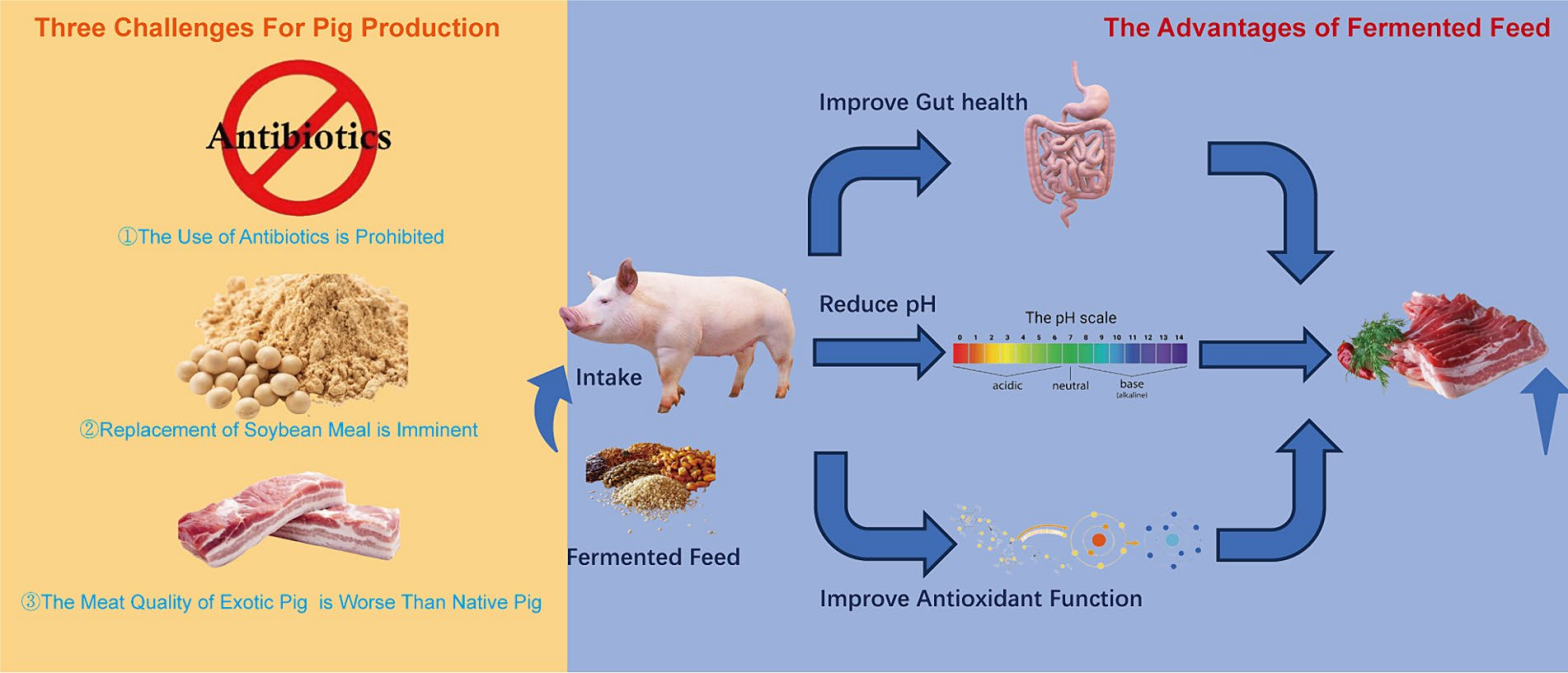
The challenge for pig production and the advantages of fermented feed.

Facing these three challenges, which include long-term reliance on soybean imports, poor meat quality of foreign pig breeds, and prohibition of antibiotic use, fermented feed presents an effective solution. It offers numerous benefits, such as enhancing the nutritional value of unconventional feedstocks (9), boosting animal immune function (10), mitigating heat stress in animals (11), improving bone quality (12), reducing harmful gases in pig housing (13), promoting intestinal health (14), and elevating pork quality (15). To address the long-term reliance on imported soybean, developing alternative protein feed sources is crucial. Unconventional protein feed materials are diverse, agri-food waste originating in various industries can be valorized for protein recovery and used in pig diets including shrimp meal, shrimp shell meal, crab meal, crab shell meal, meat and bone meal, meat meal, blood meal, fish meal, silkworm pupa meal (16). The most common unconventional protein feed resources include sesame meal, sunflower seed meal, and palm kernel meal, which are abundant in China, other countries or nearby production areas but generally suffer from poor palatability, low nutritional value, unbalanced nutrient composition, and the presence of various anti-nutritional factors (17). These problems diminish the application of unconventional protein feed materials in pig production. Numerous studies have shown that fermentation can effectively address these problems (18). Thus, the use of fermented feed is an ideal path toward high-quality pork production in China (Figure 1).

## Common types of fermented unconventional protein feeds

Protein feed is of paramount importance due to its significant role in the metabolic processes of animals (19). Fermented protein feeds are categorized into three types: plant-based, animal-based, and microbial protein feeds. High-quality conventional protein sources like soybean meal are characterized by a balanced amino acid profile and low fiber content (20). They also contain a wealth of nutrients and bioactive components such as polyphenols and phytoestrogens, which can enhance mitochondrial function and reduce oxidative stress in pigs (21). Soybean meal has become the critical source of plant protein for pigs. However, the scarcity of soybean meal resources directly leads to high production costs in the livestock industry. Thus, substituting soybean meal with unconventional protein feeds is an effective cost-saving strategy. There are many processing technologies for developing unconventional protein feed resources, such as physical techniques (e.g., grinding, puffing), chemical treatment techniques (e.g., detoxification), and biotechnologies (e.g., enzymatic hydrolysis, microbial fermentation) to improve the palatability, digestibility, and storage stability of feed ingredients (22). Fermentation technology is relatively mild and highly effective, making it the primary choice for developing unconventional protein feed resources. The general outlook for the supply of the non-conventional products in China can be seen in Table 1. This article focuses exclusively on plant-based protein feeds and investigates representative unconventional raw materials.

**Table 1:** The general outlook for the supply of non-conventional products in China.

Material	Annual	Production
Cottonseed meal	2022	4.093 million tons
Sunflower seed meal	2022	2.3 million tons
Rapeseed meal	2023	10.8 million tons
Peanut meal	2020	4.138 million tons
Peanut meal	2021	4.309 million tons
Peanut meal	2022	5.5 million tons

### Rapeseed meal

Rapeseed meal, a byproduct of oil extraction from rapeseed, contains a high crude protein content exceeding 35%. However, the presence of anti-nutritional factors such as glucosinolates, tannins, and phytic acid in rapeseed meal results in lower metabolizable energy and amino acid digestibility compared to soybean meal, and its high crude fiber content (23) limits its nutritional value in pig diets. Glucosinolates are a general term for glucose derivatives, and the content of glucosinolates in rapeseed meal is approximately 60 μmol/g (24). Glucosinolates can be hydrolyzed by enzymes, and nitrile compounds are the most toxic class of substances in the degradation products of glucosinolates, which can cause liver and kidney damage in animals, and even death in severe cases (25). Glucosinolates and their degradation products can affect the absorption and utilization of nutrients in animals by reducing animal feed intake, damaging animal thyroid and visceral organs, and producing anti-nutritional effects (26). Various methods can eliminate anti-nutritional factors in rapeseed meal, including heat treatment and solvent extraction. However, these methods may degrade the protein quality and incur high costs (27). In contrast, fermentation is a cost-effective and milder approach that can effectively remove glucosinolates and other anti-nutritional factors (28).

Among the anti-nutritional factors in rapeseed meal, tannins are generally considered unimportant. The tannins found in rapeseed meal are water-insoluble compounds located in the seed coat and have minimal impact on the nutritional value of the meal (29). In addition, moderate amounts of tannins can have beneficial effects on the productive performance and intestinal ecosystem of weaned piglets (30). Phytic acid, present at 4–6% in rapeseed meal, reduces the nutritional value of minerals such as zinc, calcium, and iron by binding with them, thus decreasing their bioavailability (31). Glucosinolates are the most toxic anti-nutritional factors in rapeseed meal, directly affecting animal thyroid function, reducing pig productivity, and consequently, pig immunity (32). Studies have shown that fermenting rapeseed meal with *Lactobacillus acidophilus* and *Bacillus subtilis* can reduce glucosinolate content from 36 μmol/g to 17 μmol/g (33). There are numerous reports on the degradation of phytic acid, detoxification of glucosinolates, and enhancement of the feed value of rapeseed meal through fermentation (34–36).

### Cottonseed meal

Cottonseed meal, a byproduct of cottonseed oil extraction, is a protein feed resource with potential in China. It has a high crude protein content, approximately 44%. However, the presence of gossypol and low lysine levels (37) greatly limit its application in pig feed. Gossypol is the primary anti-nutritional factor in cottonseed meal, a natural phenolic compound derived from cotton, predominantly found in free and bound forms in the roots, stems, leaves, and seeds of cotton plants (38). It is noteworthy that bound gossypol exhibits non-toxic characteristics due to its limited absorption in the pig’s digestive tract, while free gossypol is primarily responsible for the anti-nutritional effects in cottonseed meal (39). Free gossypol can reduce the reproductive ability of male animals, while also decreasing the antioxidant capacity of animal livers, resulting in organ damage (38). Free gossypol forms chelates with proteins, amino acids, and phospholipids in cottonseed and cottonseed meal, especially binding easily with lysine, leading to a decrease in lysine utilization (40).

Fermentation can effectively reduce the levels of free gossypol in cottonseed meal. Researchers have identified a strain with high free gossypol removal and enzymatic activity, which achieved a degradation rate of 93.46% for free gossypol in fermented cottonseed meal. This strain was identified as *B. subtilis* (41). The microbial fermentation method not only removes free gossypol from cottonseed meal but also degrades large protein molecules, thereby enhancing the nutritional value and palatability of cotton meal (42). Therefore, biofermentation is a cost-effective, efficient, and safe detoxification approach. Various microbial strains, such as *Saccharomyces cerevisiae*, *Monascus purpureus*, *B. subtilis*, and *Lactobacillus reuteri*, have been utilized to detoxify free gossypol in cottonseed meal (43, 44).

### Peanut meal

Peanut meal, a byproduct of dehulled peanuts after pre-pressing and solvent extraction for oil, is rich in bioactive substances such as peptides and polysaccharides. These components endow peanut meal with various biological functions, including antioxidant, hepatoprotective, immunomodulatory, hypolipidemic, and antimicrobial activities (45). It contains 47–55% protein, but it is deficient in essential amino acids like lysine and threonine (46). Compared to soybean meal, peanut meal has a higher energy content, but its rough texture and poor taste make it less suitable for human consumption and animal feed. Additionally, it is susceptible to mold contamination (47).

Fermentation of peanut meal can enhance its nutritional content by increasing the levels of small molecule proteins, essential amino acids, and total acids, as well as producing growth factors (48). This process not only improves the nutritional quality but also removes anti-nutritional factors and, most importantly, detoxifies mycotoxins. The anti-nutritional factors present in peanut meal include phytic acid, trypsin inhibitors, lectins, and goitrin (49). Phytic acid, with a content of approximately 1.5%, is a major factor affecting the nutritional value of peanut meal (50). Phytic acid is highly acidic and itself non-toxic or of low toxicity, its strong chelating property reduces the bioavailability of various minerals such as iron, zinc, and calcium (51). Research has identified a strain of *Zygosaccharomyces rouxii* from fermented soy sauce that, when used in aerobic solid-state fermentation of peanut meal, demonstrated a detoxification rate of up to 97% for aflatoxin B1 (52). Additionally, the use of *Bacillus licheniformis* in fermenting peanut meal has been shown to increase not only the content of organic acids and other nutritional indicators but also the antioxidant activity and the balance of amino acids (53). Current research on fermented peanut meal is primarily focused on the detoxification of mycotoxins (54).

### Palm kernel meal

Palm kernel meal, a byproduct of palm kernel oil extraction, is characterized by its abundant availability, high nutritional value of protein, low cost, and relatively low content of anti-nutritional factors (55). The protein content of palm kernel meal typically ranges from 12 to 21%, with 50.3% carbohydrates and 16.7% crude fiber (56). Although its protein content is lower compared to high-quality protein sources like soybean meal, palm kernel meal is economically priced, making it as an ideal alternative for soybean meal. Additionally, palm kernel meal has a high arginine content but low tryptophan levels. When the arginine content in feed significantly exceeds that of other amino acids, it can inhibit the digestibility of lysine. Therefore, supplementing feed with adequate lysine can help balance the feed formulation (57).

The feeding limitations of palm kernel meal are primarily due to the presence of β-mannans and fiber content. β-mannans are a type of arabinose polymer with β branches, which is not easily degraded in the animal digestive tract. It is partially soluble in water, forming a viscous substance that increases the viscosity of chyme, thereby hindering the digestion and absorption of nutrients (58). Palm kernel meal has a high crude fiber content, which leads to poor palatability of feed, resulting in reduced feed intake and metabolic capacity in animals, affecting the growth rate of animals, and is not suitable for formulating diets for piglets (59). β-mannans and fiber content are anti-nutritional factors that can be degraded through steam flashing pretreatment, multi-enzyme treatment, and microbial fermentation (60). Microbial fermentation is a common method used to reduce anti-nutritional factors and increase the utilization rate of palm kernel meal. Fermented palm kernel meal, obtained through microbial fermentation, can increase protein contents and decrease crude fiber and β-mannan content, thereby enhancing the benefits of animal husbandry (61). The high crude fiber content in palm kernel meal leads to poor palatability, mainly due to its high content of non-starch polysaccharides, which include 78% β-mannans, 12% cellulose, and 3% xylan. Additionally, palm kernel meal contains anti-nutritional factors such as galactomannan and mannan, which can increase the viscosity of intestinal contents and reduce digestibility (62). Mannans in palm kernel meal account for approximately 39% of the dry matter content, with a high content of β-branches that are highly crystalline, insoluble in water, and difficult for animals to digest and absorb (63, 64). Since animals lack enzymes to break down non-starch polysaccharides (NSP), fermentation is an effective way to degrade NSP (17).

In addition to the aforementioned feed materials, there are also many unconventional protein feed resources that can be fermented to replace conventional ingredients, including kitchen waste, flaxseed cake, palm kernel meal, coconut cake, and single-cell protein sources such as yeast.

## Factors affecting the fermentation process of unconventional protein feeds

### Temperature

Quality of fermented feed is influenced by many factors, with the most common fermentation process control factors being temperature, duration, moisture, and inoculum volume. Among these influencing factors for specific substrates and microorganisms, temperature is the most critical factor (65). The growth and metabolism of microorganisms occur under the catalysis of multiple enzymes, with temperature being an essential condition for enzyme activity. The temperature factors in the fermentation of unconventional protein feeds under different substrates and microbial strains are detailed in Table 2.

**Table 2:** Temperature factors in the fermentation of unconventional protein feeds with various substrates and microbial strains.

Substrate	Strain	Temperature	Fermentation effect	Reference
Rapeseed meal	*Bacillus subtilis*	45°C	Significant increase in acid detergent fiber	(36)
Palm kernel meal	Photosynthetic bacteria, LAB, nitrogen-fixing bacteria, yeast and *Bacillus* sp.	30–31°C	Reduction in ash content	(61)
Cottonseed meal	*Saccharomyces cerevisiae*, *Bacillus subtilis*, *Lactobacillus plantarum*	30°C	Decrease in pH	(66)
Flaxseed meal	*Bacillus subtilis*	37°C	Increase in gross energy	(67)
Feather	*Bacillus strains*	37°C	Increase in methionine	(68)
Rapeseed meal	Lactic acid bacteria	38°C	Increase in villus height of weaned piglets	(69)
Blood cells	*Aspergillus niger* and *Aspergillus oryzae*	34°C	Reduction in feed-to-gain ratio of weaned piglets	(70)

### Duration

Insufficient fermentation time may lead to failure to achieve the expected fermentation effect, while overly extended durations may result in excessive nutrient depletion and, in severe cases, autolysis of microorganisms, thereby increasing fermentation costs. The growth of microorganisms can be divided into four phases: lag phase, exponential (log) growth phase, stationary phase, and death phase. Terminating the fermentation process during the stationary phase yields the highest microbial content in the final biofeed product (71). The duration factors in the fermentation of unconventional protein feeds with various substrates and microbial strains are detailed in Table 3.

**Table 3:** Duration factors in the fermentation of unconventional protein feeds with different substrates and microbial strains.

Substrate	Strain	Time	Fermentation effect	Reference
Rapeseed meal	*Bacillus subtilis* (CICC21095)	48 h	Significant increase in neutral detergent fiber	(36)
Palm kernel meal	Photosynthetic bacteria, LAB, nitrogen-fixing bacteria, yeast and *Bacillus* sp.	30 days	Increase in crude protein	(61)
Cottonseed meal	*Saccharomyces cerevisiae, Bacillus subtilis, and Lactobacillus plantarum*	7 days	Increase in lactic acid bacteria	(66)
Flaxseed meal	*Bacillus subtilis*	14 days	Decrease in crude fat	(67)
Feather	*Bacillus strains*	2 days	Increase in lysine	(68)
Rapeseed meal	Lactic acid bacteria	4 days	Increase in weaning weight of piglets	(69)
Blood cells	*Aspergillus niger* and *Aspergillus oryzae*	116 h	Increase in average daily gain (ADG) of weaned piglets	(66)

### Moisture

Water serves as the primary medium in fermentation, acting as both a solvent and a carrier. Insufficient moisture levels can inhibit the growth and reproduction of microorganisms, while excessive moisture can lead to difficulties in heat dissipation, causing excessively high temperatures within the biomass, which may result in the death of microbial colonies (72). The moisture content of unconventional protein feed fermented with different substrates and different strains is shown in Table 4.

**Table 4:** Factors of moisture in the fermentation of unconventional protein feeds with different substrates and microbial strains.

Substrate	Strain	Moisture	Fermentation effect	Reference
Rapeseed meal	*Bacillus subtilis*	Material to water ratio of 1:0.9	Significant increase in crude protein	(36)
Flaxseed meal	*Bacillus subtilis*	Material to water ratio of 1:0.6	Decrease in crude ash	(67)
Feather	*Bacillus* strains	Material to water ratio of 1:1	Increase in crude protein	(68)

### Inoculum volume

Regarding the volume of microbial inoculum, in the case of aerobic fermentation, an excessively high inoculum volume may increase oxygen consumption, leading to a relative deficiency of oxygen supply. This can affect the normal growth and metabolism of microorganisms, resulting in waste of materials. Conversely, an insufficient inoculum volume can slow down microbial growth and reduce fermentation efficiency (73, 74). The inoculum volume factors in the fermentation of unconventional protein feeds with various substrates and microbial strains are detailed in Table 5.

**Table 5:** Factors of inoculum volume in the fermentation of unconventional protein feeds with different substrates and microbial strains.

Substrate	Strain	Inoculation amount	Fermentation effect	Reference
Feather	*Bacillus* strains	1.0 × 10^6^ CFU/g	Increase in IgA levels of fattening pigs	(68)
Palm kernel expeller	*Rhizopus oligosporus*	1.0 × 10^6^ spores per mL	Under this inoculation condition, the biomass of black soldier fly larvae increased by 34%	(60)
Peanut meal	*Bacillus licheniformis*	1.0 × 10^9^ CFU/mL	Increase in the *in vitro* digestibility rate of peanut meal	(53)
Peanut meal	*Zygosaccharomyces rouxii*	1.0 × 10^9^ CFU/mL	Significant decrease in aflatoxin B1	(52)

## Mechanisms of action of fermented unconventional protein feeds

### Growth performance

Growth performance is a critical indicator for assessing the quality of fermented feeds. Numerous studies have demonstrated that fermented unconventional protein feeds can enhance the growth performance of pigs (18, 31, 36). The mechanism by which fermented unconventional protein feeds improve pig growth performance may be attributed to their ability to increase feed intake and promote digestion and absorption.

The enhancement of feed intake in pigs by fermented unconventional protein feeds can be attributed to the production of organic acids and other appetite-stimulating substances during fermentation, particularly lactic acid produced by lactic acid bacteria, which increases feed intake in pigs (75, 76). Once these organic acids enter the gut, they can regulate the central nervous system via the gut-brain axis, thereby promoting appetite (9). Additionally, organic acids can acidify the gut and stimulate saliva secretion in pigs. The digestive enzymes in saliva, along with proteases produced by *B. subtilis* and other probiotics in the fermented feed, can break down the nutritional components in the feed, facilitating digestion and absorption (34). It is important to note that the choice of microbial strains is a key determinant of the quality of fermented feed. Different combinations of probiotics can produce varying proteolytic enzymes, affecting the absorption and digestion of nutrients (77). Therefore, from the perspective of growth performance, mixed-strain fermentation is more effective than single-strain fermentation. During fermentation, the increased activity of proteases and cellulases from probiotics leads to an increase in crude protein content and a decrease in ash, fiber, and dry matter, enhancing the nutritional value of protein feed and naturally improving pig growth performance (78). Unconventional protein feeds typically contain anti-nutritional factors such as non-starch polysaccharides, and fermentation can remove these factors and enhance nutritional value, thereby improving pig growth performance.

The digestive and absorptive efficacy of fermented feed is primarily due to the breakdown of large molecules into smaller ones during fermentation. The particle size and surface area of each substrate differ, affecting the permeability of microorganisms, with smaller molecules being more easily absorbed and digested by pigs. In the case of protein feeds, fermentation can increase the content of soluble protein and produce new peptides smaller than 25 kDa. It also breaks down large protein molecules into fragments with folded and porous surface structures, reducing the hydrophobicity and antigenicity of feed ingredients, thereby improving the properties of protein in the feed (79). Since young animals have underdeveloped digestive organs, the use of fermented feed can disrupt the physical structure of the feed. Therefore, the promotional effect of fermented feed on the growth performance and nutrient utilization rate in young animals is more pronounced (80, 81).

### Immune function

Immune function plays a crucial role in pigs, particularly in weaned piglets, who are prone to diarrhea. Influencing factors such as stress from environmental changes, loss of maternal antibodies from sow’s milk, immature immune systems, and an underdeveloped intestinal structure contribute to post-weaning diarrhea and other health issues (82). Weaning stress leads to poor nutrient absorption and reduced net absorption of electrolytes and fluids in the gut, resulting in piglet diarrhea (83). Diarrhea can cause increasing mortality of piglets and lead to a huge economic losses. Therefore, enhancing the immune function of pigs is essential. Many manufacturers use zinc oxide to prevent diarrhea in piglets, but most of the zinc oxide in feed is excreted rather than metabolized, leading to high concentrations of zinc in feces and causing environmental pollution with heavy metals (84).

Fermented feed can also improve the immune function of pigs at different stages, effectively preventing diarrhea in piglets. The mechanism behind this is the action of probiotics. Ideal probiotics used in fermentation should be resistant to gastric acid and bile salts, have the ability to colonize the gut, and possess the capability to combat pathogenic microorganisms (85). Probiotics can produce bacteriocins that inhibit the growth of harmful bacteria, activate pig macrophages, and promote the secretion of pig immunoglobulins and immune factors, such as interleukin IL-6, IL-8, tumor necrosis factor, interferons, etc., thereby enhancing the animal’s immune system. Moreover, a rich population of beneficial bacteria in the gut, along with their metabolites (lactic acid, succinic acid, short-chain fatty acids, etc.), provides an acidic environment that prevents the colonization of harmful microorganisms like *E. coli* and *Salmonella*. This also supplies an increased energy source for the gastrointestinal epithelium, promotes the development of intestinal villi, and strengthens the gut barrier function (86).

### Intestinal health

Intestinal health in pigs is closely intertwined with immune function, given that the gut is the largest immune organ in mammals and often referred to as the body’s “second brain” (87). For instance, immune responses during the weaning period in piglets can influence intestinal inflammation (88). As previously mentioned, probiotics, when consumed in adequate amounts, are live microorganisms that confer health benefits by inhibiting or eliminating pathogenic bacteria in the gut, strengthening the intestinal epithelial barrier, and modulating host immune responses (89). The intestinal epithelial barrier consists of a layer of epithelial cells that form tight structures through interactions of surface proteins, creating a barrier that blocks harmful substances and regulates the transport of nutrients. This barrier prevents the invasion of pathogenic microorganisms and food antigens, thereby maintaining consistent intestinal homeostasis (90).

Diarrhea is a prominent symptom of intestinal dysfunction, often reflecting the gut’s inability to maintain water and electrolyte balance. Weaning stress in piglets can lead to poor nutrient absorption and reduced net absorption of electrolytes and fluids in the gut, resulting in diarrhea (82). Diarrhea in weaned piglets is a significant concern in the livestock industry. Some solutions, such as porcine plasma powder, have been proposed. This product helps maintain intestinal barrier function and reduce gut inflammation when piglets transition from liquid milk to solid feed (91). An alternative, cost-effective solution is the fermentation of unconventional protein sources. Diarrhea in piglets has a multifactorial etiology, influenced by physiological, environmental, and management factors. The most common causes are pathogens, including bacteria like *E. coli* and *Salmonella*, viruses such as porcine epidemic diarrhea virus (PEDV), and parasites like nematodes and protozoa. A low pH value, an important quality indicator of fermented feed, helps inhibit the growth of harmful microorganisms and prevent spoilage (78). Probiotics from fermentation can suppress the proliferation of harmful bacteria by acidifying the gut environment and lowering the pH. Amino acids, in addition to serving as substrates for protein synthesis, also provide various beneficial effects for piglets, including maintaining proper intestinal integrity, permeability, and epithelial cell renewal, reducing morphological damage, and mitigating inflammation and oxidative stress (92). However, a high protein content in post-weaning piglet feed can increase the incidence of diarrhea, as it may active the allergic reactions and gut microbiota imbalance, with undigested proteins potentially turning into toxic substances in the hindgut (93). Fermentation reduces anti-nutritional factors and produces small molecular proteins like soluble proteins, improving amino acid digestibility and effectively preventing diarrhea (20). Insufficient fat absorption in weaned piglets can also lead to diarrhea. Factors such as carbon chain length and the ratio of unsaturated to saturated fatty acids can affect the abundance of bacteria in the piglet gut, potentially causing insufficient fat absorption and exacerbating diarrhea, as well as slowing down animal growth (94). Probiotics can regulate the absorption and metabolism of fatty acids in piglets. Fermentation can break down long-chain fatty acids into short-chain fatty acids, reducing carbon chain length and thus improving the utilization rate of fatty acid absorption (95). Studies have shown that feeding piglets with *B. licheniformis* fermented feed can have similar effects to antibiotic treatment in alleviating diarrhea (96).

Intestinal health in sows is a critical issue in the swine industry that should not be overlooked, as the gut microbiota is linked to the reproductive capacity of pregnant sows (97). The maternal gut microbiota can also be transmitted to offspring via the placenta or milk (98), thereby affecting the intestinal health of piglets. Variations in dietary nutrients can alter the composition and function of the gut microbiota in animals (99), which in turn is susceptible to temperature fluctuations (100). Lactation in sows is a period of high metabolic load, making them more sensitive to environmental temperatures. Sows subjected to heat stress often reduce their feed intake, leading to negative energy balance, loss of body condition, and reproductive issues such as anestrus, extended weaning-to-estrus interval, reduced farrowing rate, decreased litter size, and lowered milk production (101). These factors can negatively impact the growth and weaning weight of nursing piglets. To address heat stress problems in sows, plant extracts and fermented feed are proposed as the green and safe methods (102). Fermented feed can mitigate the problems caused by heat stress by modulating the intestinal health of sows. Short-chain fatty acids (SCFAs) can provide about 15% of the maintenance energy requirements for growing pigs and up to 30% for pregnant sows (103, 104). The fermentation of dietary carbohydrates primarily produces SCFAs (acetic, propionic, and butyric acids) and lactic acid as the main metabolic products, depending on the fermentable substrates and microbial ecology available in the gut (105). By altering the substrate entering the colon, the composition of SCFAs produced by the gut can be regulated. Fermentation processing can lead to increased production of SCFAs in the gut. An increase in SCFA concentrations, particularly butyric acid, improves intestinal mucosal health and the immune system in pigs (106). Therefore, fermented feed has a vital positive impact on the intestinal health of sows.

### Meat quality

The demand from Chinese livestock enterprises has primarily focused on foreign pig breeds like Duroc, Yorkshire, and Landrace due to their high reproductive performance and good economic benefits. However, compared to local pig breeds, the meat quality of these foreign white pigs is inferior, struggling to meet the evolving Chinese consumer demand from “achieving adequate” to “eating well” (107). Feeding fermented feed can effectively improve the meat quality of both pork and chicken, thus satisfying the current consumer demand for high-quality meat (81, 108, 109).

The mechanisms by which fermented unconventional protein feed improves pork quality include, but are not limited to, the following three points:

1. Fermented feed enhances pork quality by improving the antioxidant capacity in pigs. Studies have shown that an increase in the proportion of monounsaturated fatty acids and a decrease in polyunsaturated fatty acids in pork are positively correlated with the improvement of antioxidant capacity (110). There are numerous reports on the improvement of antioxidant capacity in pigs by fermented feed (14, 34, 96, 98), mainly due to the presence of probiotics that enhance antioxidant functions (111), thereby increasing the tenderness of pork.
2. Fermented feed can lower the pH value in the pig’s digestive tract, which in turn can reduce the pH of pork. The pH value of pork affects its water-holding capacity and meat color; a higher pH may lead to lighter meat color and increased water loss. The pH value of pork is related to the rate and amount of lactic acid formation in the muscle post-slaughter (112). The use of fermented feed can improve meat quality by producing lactic acid, butyric acid, and other substances.
3. The quality of pork is regulated by the gut microbiota (113), and fermented feed can improve gut health (14, 78), thereby regulating the quality of pork.

## Challenges and prospects

Despite the numerous advantages of fermented unconventional protein feeds, several challenges remain. First, contamination by adventitious microorganisms during the fermentation process is a concern; contamination can lead to the scrapping of a batch of feed, resulting in economic losses. The complexity of microorganisms may result in the production of toxic metabolites during fermentation, which can adversely affect livestock and poultry. Mycotoxins are toxic secondary metabolites produced by fungi during their growth and metabolism processes (48). For example, Aspergillus flavus produces aflatoxin B1 (AFB1) and aflatoxin B2 (AFB2) after oxidative stress stimulation from the external environment, while Fusarium produces zearalenone (ZEA) (114). Mold contamination in fermented feed mainly comes from secondary fermentation of feed during the retrieval process. The contamination details usually involve air coming into contact with the outer surface of the feed during retrieval, entering the feed, causing suppressed aerobic microorganisms (molds and yeasts, etc.) to revive, reproduce, and proliferate, with the temperature in the environment rising, further promoting microbial growth, accelerating feed decay (115). Moldy fermented feed often emits a musty odor, affecting animals’ feed intake, while molds consume valuable feed nutrients such as vitamins and amino acids, converting energy into water and CO_2_. Molds also secrete enzymes that can break down feed, significantly reducing its nutritional value. The quality of protein in fermented feed decreases, especially with a noticeable decrease in arginine and lysine content (45). Furthermore, mycotoxins can disrupt the physiological function of animals’ digestive tract, affecting the digestion and absorption of nutrients in the gastrointestinal tract, reducing animals’ productivity, endangering their health, especially liver function (52).

What’s more, the challenges of fermented feed include: Second, the safety of microbial strains is crucial, and the sourcing of strains must adhere to standardized protocols. Third, while fermented unconventional protein feeds can enhance nutritional value, the specific mechanisms are not yet fully understood. Fourth, there is a notion that mixed-strain fermentation may be more effective than single-strain fermentation, making the selection and identification of dominant strains in mixed fermentation particularly important; however, data in this area are scarce. Fifth, the issue of pelletization is continually debated; pelletization may kill probiotics due to high temperatures, while not pelletizing can lead to excessive moisture affecting feed transportation and nutrient utilization in animals (7). Sixth, improper storage methods can cause feed spoilage and increase costs.

Although fermentation of unconventional protein feeds faces many challenges, there are still several promising trends are worth to exploring in the future. Firstly, the development of a phased fermentation process that combines anaerobic and aerobic stages is an emerging trend. Secondly, fermented unconventional protein feeds play a significant role in the effective utilization of agricultural waste, greatly benefiting the rational use and development of regional feed resources. The question of how to develop regional feeds deserves attention. Thirdly, the use of liquid fermented feed signifies a transformation and upgrading in the pig farming industry. However, the practice of liquid feeding is still in its infancy, particularly regarding cost issues, making it an excellent subject for research. Fourthly, establishing a nutritional database for fermented feeds is particularly important and requires substantial investment in human and material resources to advance this work. Fifthly, replacing single fermented feeds with complete fermented feeds or concentrated fermented feeds is a trend that enterprises and research institutions should pay attention to. Lastly, strengthening scientific and technological innovation, precisely selecting efficient fermentation strains, and breeding strains through intelligent technology are essential. The development of fermented feeds requires not only the participation of professionals in animal nutrition and the fermentation industry but also the involvement of elites from other industries, such as artificial intelligence.

## Conclusion

Confronted with the challenges of soybean import dependency, inferior meat quality of imported pig breeds, and the prohibition of antibiotic use by the Ministry of Agriculture and Rural Affairs, fermentation technology emerges as a breakthrough for these issues. The rational development of unconventional protein feeds is crucial, and the fermentation of these raw materials holds broad application prospects. This paper discusses the common types of unconventional protein feeds, the fermentation processes of unconventional protein feeds, and the mechanisms by which fermented unconventional protein feeds enhance pig growth performance, immune function, and intestinal health. It summarizes the challenges faced and future trends, providing a theoretical basis for the development of protein feed resources.

## Author contributions

HS: Conceptualization, Data curation, Visualization, Writing – original draft. ZJ: Conceptualization, Visualization, Writing – original draft. ZC: Supervision, Writing – review & editing. GL: Resources, Writing – review & editing. ZL: Investigation, Writing – review & editing.

## References

[1] Wu H Liu Y Dai C Ye Y Zhu H Fang W. Life-cycle comparisons of economic and environmental consequences for pig production with four different models in China. Environ Sci Pollut Res Int. (2024) 31:21668–86. doi: 10.1007/s11356-024-32541-5 38393572

[2] Ma S Liang X Chen P Wang J Gu X Qin Y. A new single-cell protein from Clostridium autoethanogenum as a functional protein for largemouth bass (*Micropterus salmoides*). Anim Nutr. (2022) 10:99–110. doi: 10.1016/j.aninu.2022.04.005 35647322 PMC9130504

[3] Hosseindoust A Ha SH Mun JY Kim JS. A metanalysis to evaluate the effects of substrate sources on the nutritional performance of black soldier fly larvae: implications for sustainable poultry feed. Poult Sci. (2024) 103:103299. doi: 10.1016/j.psj.2023.103299 38071784 PMC10750176

[4] Chai J Zhuang Y Cui K Bi Y Zhang N. Metagenomics reveals the temporal dynamics of the rumen resistome and microbiome in goat kids. Microbiome. (2024) 12:14. doi: 10.1186/s40168-023-01733-5 38254181 PMC10801991

[5] Wu Z Liu J Chen J Pirzado SA Li Y Cai H. Effects of fermentation on standardized ileal digestibility of amino acids and apparent metabolizable energy in rapeseed meal fed to broiler chickens. Animals. (2020) 10:1774. doi: 10.3390/ani10101774 33019513 PMC7599665

[6] Xu B Fu J Zhu L Li Z Jin M Wang Y. Overall assessment of antibiotic substitutes for pigs: a set of meta-analyses. J Anim Sci Biotechnol. (2021) 12:3. doi: 10.1186/s40104-020-00534-2 33413687 PMC7792336

[7] Feng J Lu M Wang J Zhang H Qiu K Qi G. Dietary oregano essential oil supplementation improves intestinal functions and alters gut microbiota in late-phase laying hens. J Anim Sci Biotechnol. (2021) 12:72. doi: 10.1186/s40104-021-00600-3 34225796 PMC8259136

[8] Ricke SC Lee SI Kim SA Park SH Shi Z. Prebiotics and the poultry gastrointestinal tract microbiome. Poult Sci. (2020) 99:670–7. doi: 10.1016/j.psj.2019.12.018 32029153 PMC7587714

[9] Sun H Kang X Tan H Cai H Chen D. Progress in fermented unconventional feed application in monogastric animal production in China. Fermentation. (2023) 9:947. doi: 10.3390/fermentation9110947

[10] Mizumachi K Aoki R Ohmori H Saeki M Kawashima T. Effect of fermented liquid diet prepared with *Lactobacillus plantarum* LQ80 on the immune response in weaning pigs. Animal. (2009) 3:670–6. doi: 10.1017/S1751731109003978 22444444

[11] Qiu Y Zhao H He X Zhu F Zhang F Liu B. Effects of fermented feed of Pennisetum giganteum on growth performance, oxidative stress, immunity and gastrointestinal microflora of Boer goats under thermal stress. Front Microbiol. (2022) 13:1030262. doi: 10.3389/fmicb.2022.1030262 36713179 PMC9879058

[12] Zhang H Wang Y Wang Y Wei B Wang L Nguyen MT. Fermented calcium butyrate supplementation in post-peak laying hens improved ovarian function and tibia quality through the “gut-bone” axis. Anim Nutr. (2024) 16:350–62. doi: 10.1016/j.aninu.2023.10.008 38362518 PMC10867563

[13] Hossain MM Cho SB Kim IH. Strategies for reducing noxious gas emissions in pig production: a comprehensive review on the role of feed additives. J Anim Sci Technol. (2024) 66:237–50. doi: 10.5187/jast.2024.e15 38628679 PMC11016746

[14] Su W Jiang Z Wang C Zhang Y Gong T Wang F. Co-fermented defatted rice bran alters gut microbiota and improves growth performance, antioxidant capacity, immune status and intestinal permeability of finishing pigs. Anim Nutr. (2022) 11:413–24. doi: 10.1016/j.aninu.2022.07.008 36382202 PMC9640948

[15] Tian Z Deng D Cui Y Chen W Yu M Ma X. Diet supplemented with fermented okara improved growth performance, meat quality, and amino acid profiles in growing pigs. Food Sci Nutr. (2020) 8:5650–9. doi: 10.1002/fsn3.1857 33133567 PMC7590273

[16] Kim SW Less JF Wang L Yan T Kiron V Kaushik SJ. Meeting global feed protein demand: challenge, opportunity, and strategy. Annu Rev Anim Biosci. (2019) 7:221–43. doi: 10.1146/annurev-animal-030117-014838 30418803

[17] Olukomaiya O Fernando C Mereddy R Li X Sultanbawa Y. Solid-state fermented plant protein sources in the diets of broiler chickens: a review. Anim Nutr. (2019) 5:319–30. doi: 10.1016/j.aninu.2019.05.005 31890908 PMC6920459

[18] Xu B Zhu L Fu J Li Z Wang Y Jin M. Overall assessment of fermented feed for pigs: a series of meta-analyses. J Anim Sci. (2019) 97:4810–21. doi: 10.1093/jas/skz350 31712812 PMC6915212

[19] Beski SSM Swick RA Iji PA. Specialized protein products in broiler chicken nutrition: a review. Anim Nutr. (2015) 1:47–53. doi: 10.1016/j.aninu.2015.05.005 29766993 PMC5884466

[20] Jeong JS Park JW Lee SI Kim IH. Apparent ileal digestibility of nutrients and amino acids in soybean meal, fish meal, spray-dried plasma protein and fermented soybean meal to weaned pigs. Anim Sci J. (2016) 87:697–702. doi: 10.1111/asj.12483 26300306

[21] Hemler EC Tamez M Orengo JFR Mattei J. Positive attitudes toward legumes are associated with legume intake among adults in Puerto Rico. Nutr Res. (2022) 103:21–9. doi: 10.1016/j.nutres.2022.03.006 35453043 PMC9156569

[22] Kiarie EG Mills A. Role of feed processing on gut health and function in pigs and poultry: conundrum of optimal particle size and hydrothermal regimens. Front Vet Sci. (2019) 6:19. doi: 10.3389/fvets.2019.00019 30838217 PMC6390496

[23] Zhou B Ran H Zhang Q Chen H Han F Xu C. Unveiling the impact of rapeseed meal on feeding behavior and anorexigenic endocrine in *Litopenaeus vannamei*. Animals. (2024) 14:540. doi: 10.3390/ani14040540 38396508 PMC10886117

[24] Seneviratne RW Beltranena E Newkirk RW Goonewardene LA Zijlstra RT. Processing conditions affect nutrient digestibility of cold-pressed canola cake for grower pigs. J Anim Sci. (2011) 89:2452–61. doi: 10.2527/jas.2010-3569 21421830

[25] Grubb CD Abel S. Glucosinolate metabolism and its control. Trends Plant Sci. (2006) 11:89–100. doi: 10.1016/j.tplants.2005.12.006 16406306

[26] Ullah Z Rehman ZU Yin Y Stein HH Hayat Z Ahmed G. Comparative ileal digestibility of amino acids in 00-rapeseed meal and rapeseed meal fed to growing male broilers. Poult Sci. (2017) 96:2736–42. doi: 10.3382/ps/pex083 28453657

[27] Konkol D Popiela E Opalinski S Lipinska A Tymoszewski A Krasowska A. Effects of fermented rapeseed meal on performance, intestinal morphology, the viscosity of intestinal content, phosphorus availability, and egg quality of laying hens. Poult Sci. (2024) 103:103256. doi: 10.1016/j.psj.2023.103256 37980734 PMC10684812

[28] Xu FZ Zeng XG Ding XL. Effects of replacing soybean meal with fermented rapeseed meal on performance, serum biochemical variables and intestinal morphology of broilers. Asian Australas J Anim Sci. (2012) 25:1734–41. doi: 10.5713/ajas.2012.12249 25049539 PMC4094158

[29] Vlassa M Filip M Taranu I Marin D Untea AE Ropota M. The yeast fermentation effect on content of bioactive, nutritional and anti-nutritional factors in rapeseed meal. Food Secur. (2022) 11:2972. doi: 10.3390/foods11192972 PMC9562236 36230048

[30] Mejicanos G Sanjayan N Kim IH Nyachoti CM. Recent advances in canola meal utilization in swine nutrition. J Anim Sci Technol. (2016) 58:7. doi: 10.1186/s40781-016-0085-5 26885377 PMC4754856

[31] Choi HB Jeong JH Kim DH Lee Y Kwon H Kim YY. Influence of rapeseed meal on growth performance, blood profiles, nutrient digestibility and economic benefit of growing-finishing pigs. Asian Australas J Anim Sci. (2015) 28:1345–53. doi: 10.5713/ajas.14.0802 26323520 PMC4554876

[32] Frandsen HB Sorensen JC Jensen SK Markedal KE Joehnke MS Maribo H. Non-enzymatic transformations of dietary 2-hydroxyalkenyl and aromatic glucosinolates in the stomach of monogastrics. Food Chem. (2019) 291:77–86. doi: 10.1016/j.foodchem.2019.03.136 31006474

[33] Wu S Wang J Zhu L Ren H Yang X. A novel apidaecin Api-PR19 synergizes with the gut microbial community to maintain intestinal health and promote growth performance of broilers. J Anim Sci Biotechnol. (2020) 11:61. doi: 10.1186/s40104-020-00462-1 32551109 PMC7298829

[34] Czech A Nowakowicz-Debek B Lukaszewicz M Florek M Ossowski M Wlazlo L. Effect of fermented rapeseed meal in the mixture for growing pigs on the gastrointestinal tract, antioxidant status, and immune response. Sci Rep. (2022) 12:15764. doi: 10.1038/s41598-022-20227-2 36130989 PMC9492901

[35] Grela ER Czech A Kiesz M Wlazlo L Nowakowicz-Debek B. A fermented rapeseed meal additive: effects on production performance, nutrient digestibility, colostrum immunoglobulin content and microbial flora in sows. Anim Nutr. (2019) 5:373–9. doi: 10.1016/j.aninu.2019.05.004 31890914 PMC6920389

[36] Shuai C Chen D Yu B Luo Y Zheng P Huang Z. Effect of fermented rapeseed meal on growth performance, nutrient digestibility, and intestinal health in growing pigs. Anim Nutr. (2023) 15:420–9. doi: 10.1016/j.aninu.2023.06.011 38058565 PMC10696392

[37] Lv L Xiong F Liu Y Pei S He S Li S. The rumen-derived Lact. mucosae LLK-XR1 exhibited greater free gossypol degradation capacity during solid-state fermentation of cottonseed meal and probiotic potential. BMC Microbiol. (2024) 24:15. doi: 10.1186/s12866-023-03156-6 38183000 PMC10768434

[38] Gadelha IC Fonseca NB Oloris SC Melo MM Soto-Blanco B. Gossypol toxicity from cottonseed products. Sci World J. (2014) 2014:231635. doi: 10.1155/2014/231635 PMC4033412 24895646

[39] EFSA Panel on Contaminants in the Food Chain (CONTAM) Knutsen HK Barregard L Bignami M Bruschweiler B Ceccatelli S. Presence of free gossypol in whole cottonseed. EFSA J. (2017) 15:e04850. doi: 10.2903/j.efsa.2017.4850, 32625538 PMC7010194

[40] Wang X Howell CP Chen F Yin J Jiang Y. Gossypol--a polyphenolic compound from cotton plant. Adv Food Nutr Res. (2009) 58:215–63. doi: 10.1016/S1043-4526(09)58006-0 19878861

[41] Li J Gao T Hao Z Guo X Zhu B. Anaerobic solid-state fermentation with *Bacillus subtilis* for digesting free gossypol and improving nutritional quality in cottonseed meal. Front Nutr. (2022) 9:1017637. doi: 10.3389/fnut.2022.1017637 36570163 PMC9773203

[42] Niu JL Wei LQ Luo YQ Yang WT Lu QC Zheng XX. Fermented cottonseed meal improves production performance and reduces fat deposition in broiler chickens. Anim Biosci. (2021) 34:680–91. doi: 10.5713/ajas.20.0571 33254361 PMC7961297

[43] Xie SS Shen JJ Liu Y Yang ZL Wang WC Yang L. Effects of fermented cottonseed meal inclusions on growth performance, serum biochemical parameters and hepatic lipid metabolism of geese during 28-70 d of age. Poult Sci. (2024) 103:103702. doi: 10.1016/j.psj.2024.103702 38652950 PMC11063510

[44] Zhang Z Yang D Liu L Chang Z Peng N. Effective gossypol removal from cottonseed meal through optimized solid-state fermentation by *Bacillus coagulans*. Microb Cell Factories. (2022) 21:252. doi: 10.1186/s12934-022-01976-1 PMC9714218 36456988

[45] Zhao T Ying P Zhang Y Chen H Yang X. Research advances in the high-value utilization of peanut meal resources and its hydrolysates: a review. Molecules. (2023) 28:6862. doi: 10.3390/molecules28196862 37836705 PMC10574612

[46] Li X Rezaei R Li P Wu G. Composition of amino acids in feed ingredients for animal diets. Amino Acids. (2011) 40:1159–68. doi: 10.1007/s00726-010-0740-y 20842395

[47] Xia WG Abouelezz KFM Makled MN Wang S Chen W Zhang YN. Effects of dietary substitution of peanut meal for soybean meal on egg production, egg quality, oxidative status, and yolk fatty acid profile in laying ducks. Animal. (2022) 16:100652. doi: 10.1016/j.animal.2022.100652 36265190

[48] Lu Y Ding H Jiang X Zhang H Ma A Hu Y. Effects of the extract from peanut meal fermented with Bacillus natto and Monascus on lipid metabolism and intestinal barrier function of hyperlipidemic mice. J Sci Food Agric. (2021) 101:2561–9. doi: 10.1002/jsfa.10884 33063356

[49] Dias CAS Bagaldo AR Cerutti WG Barbosa AM De Carvalho GGP Costa EIS. Peanut cake can replace soybean meal in supplements for lactating cows without affecting production. Trop Anim Health Prod. (2018) 50:651–7. doi: 10.1007/s11250-017-1482-6 29238885

[50] Zhao X Chen J Du F. Potential use of peanut by-products in food processing: a review. J Food Sci Technol. (2012) 49:521–9. doi: 10.1007/s13197-011-0449-2 24082262 PMC3550843

[51] Anekonda TS Wadsworth TL Sabin R Frahler K Harris C Petriko B. Phytic acid as a potential treatment for Alzheimer’s pathology: evidence from animal and in vitro models. J Alzheimers Dis. (2011) 23:21–35. doi: 10.3233/JAD-2010-101287 20930278 PMC3021000

[52] Zhou G Chen Y Kong Q Ma Y Liu Y. Detoxification of aflatoxin B₁ by *Zygosaccharomyces rouxii* with solid state fermentation in peanut meal. Toxins. (2017) 9:42. doi: 10.3390/toxins9010042 28117705 PMC5308274

[53] Yang X Teng D Wang X Guan Q Mao R Hao Y. Enhancement of nutritional and antioxidant properties of peanut meal by bio-modification with *Bacillus licheniformis*. Appl Biochem Biotechnol. (2016) 180:1227–42. doi: 10.1007/s12010-016-2163-z 27318709

[54] Fan Y Zhao L Ma Q Li X Shi H Zhou T. Effects of *Bacillus subtilis* ANSB060 on growth performance, meat quality and aflatoxin residues in broilers fed moldy peanut meal naturally contaminated with aflatoxins. Food Chem Toxicol. (2013) 59:748–53. doi: 10.1016/j.fct.2013.07.010 23872125

[55] Wang T Li S Ning J Li J Han Y Yin X. Effects of different processing techniques of palm kernel cake on processing quality of pellet feed, nutrient digestibility, and intestinal microbiota of pigs. J Anim Sci. (2023) 101:skad217. doi: 10.1093/jas/skad217 37357763 PMC10362929

[56] Bisinotto MS Da Silva Napoli DC Simabuco FM Bezerra RMN Antunes AEC Galland F. Sunflower and palm kernel meal present bioaccessible compounds after digestion with antioxidant activity. Food Secur. (2023) 12:3283. doi: 10.3390/foods12173283 PMC10486993 37685216

[57] Ma Y Han L Raza SHA Gui L Zhang X Hou S. Exploring the effects of palm kernel meal feeding on the meat quality and rumen microorganisms of Qinghai Tibetan sheep. Food Sci Nutr. (2023) 11:3516–34. doi: 10.1002/fsn3.3340 37324863 PMC10261763

[58] Behan JL Smith KD. The analysis of glycosylation: a continued need for high pH anion exchange chromatography. Biomed Chromatogr. (2011) 25:39–46. doi: 10.1002/bmc.1514 20821735

[59] Chen WL Tang SGH Jahromi MF Candyrine SCL Idrus Z Abdullah N. Metagenomics analysis reveals significant modulation of cecal microbiota of broilers fed palm kernel expeller diets. Poult Sci. (2019) 98:56–68. doi: 10.3382/ps/pey366 30137571

[60] Liew CS Wong CY Abdelfattah EA Raksasat R Rawindran H Lim JW. Fungal fermented palm kernel expeller as feed for black soldier fly larvae in producing protein and biodiesel. J Fungi. (2022) 8:332. doi: 10.3390/jof8040332 PMC9025283 35448563

[61] Wattanakul W Thongprajukaew K Hahor W Suanyuk N. Optimal replacement of soybean meal with fermented palm kernel meal as protein source in a fish meal-soybean meal-based diet of sex reversed red tilapia (*Oreochromis niloticus* × *O. mossambicus*). Animals. (2021) 11:2287. doi: 10.3390/ani11082287 34438745 PMC8388480

[62] Yılmaz C Gökmen V. Formation of amino acid derivatives in white and red wines during fermentation: effects of non-Saccharomyces yeasts and *Oenococcus oeni*. Food Chem. (2021) 343:128415. doi: 10.1016/j.foodchem.2020.128415 33268169

[63] Choi WJ Kim JH Kim HW Kim KE Kil DY. Effects of dietary palm kernel meal and β-xylanase on productive performance, fatty liver incidence, and excreta characteristics in laying hens. J Anim Sci Technol. (2021) 63:1275–85. doi: 10.5187/jast.2021.e111 34957443 PMC8672254

[64] Khophloiklang V Chanapiwat P Aunpad R Kaeoket K. Palm kernel meal protein hydrolysates enhance post-thawed boar sperm quality. Animals. (2023) 13:3040. doi: 10.3390/ani13193040 37835646 PMC10571854

[65] Yang L Zeng X Qiao S. Advances in research on solid-state fermented feed and its utilization: the pioneer of private customization for intestinal microorganisms. Anim Nutr. (2021) 7:905–16. doi: 10.1016/j.aninu.2021.06.002 34632121 PMC8482288

[66] Ashayerizadeh A Jazi V Rezvani MR Mohebodini H Soumeh EA Abdollahi MR. An investigation into the influence of fermented cottonseed meal on the productive performance, egg quality, and gut health in laying hens. Poult Sci. (2024) 103:103574. doi: 10.1016/j.psj.2024.103574 38564832 PMC10999706

[67] Wei Z Xu L Guo Y Guo B Lu C Sun W. Evaluation of available energy and standardized Ileal digestibility of amino acids in fermented flaxseed meal for growing pigs. Animals. (2024) 14:228. doi: 10.3390/ani14020228 38254397 PMC10812548

[68] Huang HJ Weng BC Hsuuw YD Lee YS Chen KL. Dietary supplementation of two-stage fermented feather-soybean meal product on growth performance and immunity in finishing pigs. Animals. (2021) 11:1527. doi: 10.3390/ani11061527 34073850 PMC8225001

[69] Satessa GD Tamez-Hidalgo P Hui Y Cieplak T Krych L Kjaerulff S. Impact of dietary supplementation of lactic acid bacteria fermented rapeseed with or without macroalgae on performance and health of piglets following omission of medicinal zinc from weaner diets. Animals. (2020) 10:137. doi: 10.3390/ani10010137 31952154 PMC7023219

[70] Zhang B Yang W Zhang H Meng Q Bi C Shan A. Effect of fermented blood cells on growth performance and intestinal characteristics of weaned piglets. J Anim Physiol Anim Nutr. (2019) 103:1875–84. doi: 10.1111/jpn.13194 31483538

[71] Graham AE Ledesma-Amaro R. The microbial food revolution. Nat Commun. (2023) 14:2231. doi: 10.1038/s41467-023-37891-1 37076544 PMC10115867

[72] De Roos J De Vuyst L. Acetic acid bacteria in fermented foods and beverages. Curr Opin Biotechnol. (2018) 49:115–9. doi: 10.1016/j.copbio.2017.08.007 28863341

[73] Missotten JA Michiels J Ovyn A De Smet S Dierick NA. Fermented liquid feed for pigs. Arch Anim Nutr. (2010) 64:437–66. doi: 10.1080/1745039X.2010.512725 21214019

[74] Plumed-Ferrer C Von Wright A. Fermented pig liquid feed: nutritional, safety and regulatory aspects. J Appl Microbiol. (2009) 106:351–68. doi: 10.1111/j.1365-2672.2008.03938.x 19016978

[75] Chen H Heng X Li K Wang Z Ni Z Gao E. Complexation of multiple mineral elements by fermentation and its application in laying hens. Front Nutr. (2022) 9:1001412. doi: 10.3389/fnut.2022.1001412 36245477 PMC9556719

[76] Liu J Wang K Zhao L Li Y Li Z Li C. Investigation of supplementation with a combination of fermented bean dregs and wheat bran for improving the growth performance of the sow. J Anim Sci Technol. (2024) 66:295–309. doi: 10.5187/jast.2023.e13 38628686 PMC11016735

[77] Chen X Wu C Li X Wang C Li Q Zhou P. Effect of *Geobacillus toebii* GT-02 addition on composition transformations and microbial community during thermophilic fermentation of bean dregs. Sci Rep. (2021) 11:19949. doi: 10.1038/s41598-021-99413-7 34620903 PMC8497473

[78] Zhang M Yang Z Wu G Xu F Zhang J Luo X. Effects of probiotic-fermented feed on the growth profile, immune functions, and intestinal microbiota of Bamei piglets. Animals. (2024) 14:647. doi: 10.3390/ani14040647 38396614 PMC10886304

[79] Miao X Niu H Sun M Li D Hua M Wang J. Structural characterization and properties of modified soybean meal protein via solid-state fermentation by *Bacillus subtilis*. Molecules. (2023) 28:8015. doi: 10.3390/molecules28248015 38138505 PMC10746062

[80] Qiu Y Tang J Wang L Yang X Jiang Z. Fermented corn-soybean meal improved growth performance and reduced diarrhea incidence by modulating intestinal barrier function and gut microbiota in weaned piglets. Int J Mol Sci. (2024) 25:3199. doi: 10.3390/ijms25063199 38542173 PMC10970572

[81] Sun H Chen D Cai H Chang W Wang Z Liu G. Effects of fermenting the plant fraction of a complete feed on the growth performance, nutrient utilization, antioxidant functions, meat quality, and intestinal microbiota of broilers. Animals. (2022) 12:2870. doi: 10.3390/ani12202870 36290256 PMC9597820

[82] Jenkins TP Acs N Arendrup EW Swift A Duzs A Chatzigiannidou I. Protecting the piglet gut microbiota against ETEC-mediated post-weaning diarrhoea using specific binding proteins. NPJ Biofi Microb. (2024) 10:42. doi: 10.1038/s41522-024-00514-8 PMC11066037 38697985

[83] Xia J Fan H Yang J Song T Pang L Deng H. Research progress on diarrhoea and its mechanism in weaned piglets fed a high-protein diet. J Anim Physiol Anim Nutr. (2022) 106:1277–87. doi: 10.1111/jpn.13654 34719816

[84] Jensen J Kyvsgaard NC Battisti A Baptiste KE. Environmental and public health related risk of veterinary zinc in pig production – using Denmark as an example. Environ Int. (2018) 114:181–90. doi: 10.1016/j.envint.2018.02.007 29518661

[85] Wang L Wang C Peng Y Zhang Y Liu Y Liu Y. Research progress on anti-stress nutrition strategies in swine. Anim Nutr. (2023) 13:342–60. doi: 10.1016/j.aninu.2023.03.006 37214213 PMC10192683

[86] Yao T Wang C Liang L Xiang X Zhou H Zhou W. Effects of fermented sweet potato residue on nutrient digestibility, meat quality, and intestinal microbes in broilers. Anim Nutr. (2024) 17:75–86. doi: 10.1016/j.aninu.2024.03.007 38737580 PMC11087712

[87] Xiong X Tan B Song M Ji P Kim K Yin Y. Nutritional intervention for the intestinal development and health of weaned pigs. Front Vet Sci. (2019) 6:46. doi: 10.3389/fvets.2019.00046 30847348 PMC6393345

[88] Tang W Wei Y Ni Z Hou K Luo XM Wang H. IgA-mediated control of host-microbial interaction during weaning reaction influences gut inflammation. Gut Micr. (2024) 16:2323220. doi: 10.1080/19490976.2024.2323220 PMC10936605 38439579

[89] Abrehame S Hung MY Chen YY Liu YT Chen YT Liu FC. Selection of fermentation supernatant from probiotic strains exhibiting intestinal epithelial barrier protective ability and evaluation of their effects on colitis mouse and weaned piglet models. Nutrients. (2024) 16:1138. doi: 10.3390/nu16081138 38674829 PMC11053620

[90] De Vos WM Tilg H Van Hul M Cani PD. Gut microbiome and health: mechanistic insights. Gut. (2022) 71:1020–32. doi: 10.1136/gutjnl-2021-326789 35105664 PMC8995832

[91] Kazimierska K Biel W. Chemical composition and functional properties of spray-dried animal plasma and its contributions to livestock and pet health: a review. Animals. (2023) 13:2484. doi: 10.3390/ani13152484 37570293 PMC10416976

[92] Zhou X Liang J Xiong X Yin Y. Amino acids in piglet diarrhea: effects, mechanisms and insights. Anim Nutr. (2024) 16:267–74. doi: 10.1016/j.aninu.2023.07.009 38362520 PMC10867606

[93] Kiers JL Meijer JC Nout MJ Rombouts FM Nabuurs MJ Van Der Meulen J. Effect of fermented soya beans on diarrhoea and feed efficiency in weaned piglets. J Appl Microbiol. (2003) 95:545–52. doi: 10.1046/j.1365-2672.2003.02011.x 12911703

[94] Li Y Shi P Yao K Lin Q Wang M Hou Z. Diarrhea induced by insufficient fat absorption in weaned piglets: causes and nutrition regulation. Anim Nutr. (2024) 16:299–305. doi: 10.1016/j.aninu.2023.12.004 38371473 PMC10869582

[95] Xu E Chen C Fu J Zhu L Shu J Jin M. Dietary fatty acids in gut health: absorption, metabolism and function. Anim Nutr. (2021) 7:1337–44. doi: 10.1016/j.aninu.2021.09.010 34786506 PMC8570925

[96] Lin KH Yu YH. Evaluation of *Bacillus licheniformis*-fermented feed additive as an antibiotic substitute: effect on the growth performance, diarrhea incidence, and Cecal microbiota in weaning piglets. Animals. (2020) 10:1649. doi: 10.3390/ani10091649 32937883 PMC7552216

[97] Ye Q Luo T Han L Chen Y Hu Y Jiang H. Multi-omics analysis reveals the dominant intestinal microbial strains and metabolites related to the reproductive performance in pregnant sows. Anim Nutr. (2024) 1:1–33. doi: 10.1017/anr.2024.7

[98] Zhang Y Deng Y Hao Y Fang J Feng J. Effects of supplementation with oregano essential oil during late gestation and lactation on serum metabolites, antioxidant capacity and fecal microbiota of sows. Animals. (2024) 14:753. doi: 10.3390/ani14050753 38473138 PMC10931315

[99] Olson EG Dittoe DK Jendza JA Stock DA Ricke SC. Application of microbial analyses to feeds and potential implications for poultry nutrition. Poult Sci. (2022) 101:101789. doi: 10.1016/j.psj.2022.101789 35346494 PMC9079344

[100] Hakim AH Zulkifli I Farjam AS Awad EA Ramiah SK. Impact of feeding fermented palm kernel cake and high dietary fat on nutrient digestibility, enzyme activity, intestinal morphology and intestinal nutrient transporters mRNA expression in broiler chickens under hot and humid conditions. Animals. (2022) 12:882. doi: 10.3390/ani12070882 35405871 PMC8997065

[101] Lucy MC Safranski TJ. Heat stress in pregnant sows: thermal responses and subsequent performance of sows and their offspring. Mol Reprod Dev. (2017) 84:946–56. doi: 10.1002/mrd.22844 28696547

[102] Wen C Wei S Zong X Wang Y Jin M. Microbiota-gut-brain axis and nutritional strategy under heat stress. Anim Nutr. (2021) 7:1329–36. doi: 10.1016/j.aninu.2021.09.008 34786505 PMC8570956

[103] Jiao A Diao H Yu B He J Yu J Zheng P. Infusion of short chain fatty acids in the ileum improves the carcass traits, meat quality and lipid metabolism of growing pigs. Anim Nutr. (2021) 7:94–100. doi: 10.1016/j.aninu.2020.05.009 33997336 PMC8110845

[104] Zhou J Xiong X Yin J Zou L Wang K Shao Y. Dietary lysozyme alters sow’s gut microbiota, serum immunity and milk metabolite profile. Front Microbiol. (2019) 10:177. doi: 10.3389/fmicb.2019.00177 30787921 PMC6373202

[105] Tian M Chen J Liu J Chen F Guan W Zhang S. Dietary fiber and microbiota interaction regulates sow metabolism and reproductive performance. Anim Nutr. (2020) 6:397–403. doi: 10.1016/j.aninu.2020.10.001 33364455 PMC7750804

[106] Tiwari UP Singh AK Jha R. Fermentation characteristics of resistant starch, arabinoxylan, and beta-glucan and their effects on the gut microbial ecology of pigs: a review. Anim Nutr. (2019) 5:217–26. doi: 10.1016/j.aninu.2019.04.003 31528722 PMC6737498

[107] Liu H He J Yuan Z Xie K He Z Zhou X. Metabolomics analysis provides novel insights into the difference in meat quality between different pig breeds. Food Secur. (2023) 12:3476. doi: 10.3390/foods12183476 PMC10528157 37761184

[108] Jeong H Lee S Han GD. The effect of sigumjang (Korean fermented barley bran) marination on the physicochemical properties of pork loin. Food Sci Biotechnol. (2020) 29:1195–9. doi: 10.1007/s10068-020-00767-x 32802558 PMC7406602

[109] Qiu Y Li K Zhao X Liu S Wang L Yang X. Fermented feed modulates meat quality and promotes the growth of longissimus Thoracis of late-finishing pigs. Animals. (2020) 10:1682. doi: 10.3390/ani10091682 32957692 PMC7552782

[110] Han M Yin Y Gong S Shi H Li Q Lian X. Effects of dietary *Eucommia ulmoides* leaf extract supplementation on growth performance, meat quality, antioxidant capacity, and lipid metabolism of finishing pigs. Antioxidants. (2024) 13:320. doi: 10.3390/antiox13030320 38539852 PMC10967616

[111] Jiang Z Su W Li W Wen C Du S He H. *Bacillus amyloliquefaciens* 40 regulates piglet performance, antioxidant capacity, immune status and gut microbiota. Anim Nutr. (2023) 12:116–27. doi: 10.1016/j.aninu.2022.09.006 36632621 PMC9826887

[112] Ludwiczak A Kasprowicz-Potocka M Zaworska-Zakrzewska A Skladanowska-Baryza J Rodriguez-Estevez V Sanz-Fernandez S. Husbandry practices associated with extensification in European pig production and their effects on pork quality. Meat Sci. (2023) 206:109339. doi: 10.1016/j.meatsci.2023.109339 37716226

[113] Hou J Ji X Chu X Wang B Sun K Wei H. Mulberry leaf dietary supplementation can improve the lipo-nutritional quality of pork and regulate gut microbiota in pigs: a comprehensive multi-omics analysis. Animals. (2024) 14:1233. doi: 10.3390/ani14081233 38672381 PMC11047539

[114] Chen Y Kong Q Chi C Shan S Guan B. Biotransformation of aflatoxin B1 and aflatoxin G1 in peanut meal by anaerobic solid fermentation of Streptococcus thermophilus and *Lactobacillus delbrueckii* subsp. bulgaricus. Int J Food Microbiol. (2015) 211:1–5. doi: 10.1016/j.ijfoodmicro.2015.06.021 26143229

[115] Liu M Zhao L Gong G Zhang L Shi L Dai J. Invited review: remediation strategies for mycotoxin control in feed. J Anim Sci Biotechnol. (2022) 13:19. doi: 10.1186/s40104-021-00661-4 35090579 PMC8796454

